# Increased expression of long noncoding RNA TUG1 predicts a poor prognosis of gastric cancer and regulates cell proliferation by epigenetically silencing of p57

**DOI:** 10.1038/cddis.2015.356

**Published:** 2016-02-25

**Authors:** E Zhang, X He, D Yin, L Han, M Qiu, T Xu, R Xia, L Xu, R Yin, W De

**Affiliations:** 1 Department of Biochemistry and Molecular Biology, Nanjing Medical University, Nanjing, Jiangsu, China; 2 Central Laboratory, The Second Affiliated Hospital of Southeast University, Nanjing, Jiangsu, China; 3 Department of Oncology, Xuzhou Central Hospital, Affiliated Xuzhou Hospital, College of Medicine, Southeast University, Xuzhou, Jiangsu, China; 4 Department of Thoracic Surgery, Jiangsu Key Laboratory of Molecular and Translational Cancer Research, Nanjing Medical University Affiliated Cancer Hospital, Cancer Institute of Jiangsu Province, Nanjing, Jiangsu, China; 5 Department of Oncology, First Affiliated Hospital of Nanjing Medical University, Nanjing, Jiangsu, China

## Abstract

Recent evidence highlights long noncoding RNAs (lncRNAs) as crucial regulators of cancer biology that contribute to tumorigenesis. LncRNA TUG1 was initially detected in a genomic screen for genes upregulated in response to taurine treatment in developing mouse retinal cells. Our previous study showed that TUG1 could affect cell proliferation through epigenetically regulating HOXB7 in human non-small cell lung cancer. However, the clinical significance and potential role of TUG1 in GC remains unclear. In this study, we found that TUG1 is significantly increased and is correlated with outcomes in gastric cancer (GC). Further experiments revealed that knockdown of TUG1 repressed GC proliferation both *in vitro* and *in vivo*. Mechanistic investigations showed that TUG1 has a key role in G0/G1 arrest. We further demonstrated that TUG1 was associated with PRC2 and that this association was required for epigenetic repression of cyclin-dependent protein kinase inhibitors, including p15, p16, p21, p27 and p57, thus contributing to the regulation of GC cell cycle and proliferation. Together, our results suggest that TUG1, as a regulator of proliferation, may serve as a candidate prognostic biomarker and target for new therapies in human GC.

Gastric cancer (GC) is one of the most common malignancies worldwide.^1^ Despite efforts to improve diagnostic techniques and patient management, there has been little progress toward improving the overall survival of GC patients.^2^ Gastric carcinogenesis is a complicated biological process, which results from the dysregulation of many tumor-related genes. Therefore, the identification of new biomarkers for GC and a better understanding of the molecular mechanisms underlying gastric carcinogenesis will improve the diagnosis and treatment of GC.

With the development of next-generation sequencing technologies, it was determined that long noncoding RNAs (lncRNAs) are pervasively transcribed in the genome.^3, 4^ LncRNAs are a class of transcripts longer than 200 nucleotides with limited protein-coding potential.^5^ Recently, many studies have shown that lncRNAs could have critical roles in many biological processes including cellular development and differentiation.^6, 7, 8, 9, 10^ The aberrant expression of lncRNAs has also been shown in various types of disease, including cancer.^11, 12, 13, 14, 15, 16^ For example, HOTAIR may be involved in the transcriptional repression of the HOX loci and promote breast metastasis by binding to polycomb repressive complex 2 (PRC2).^13^ In addition to regulation at the transcriptional level, lncRNAs can also serve as a ‘sponge' to titrate microRNAs, thus participating in post-transcriptional processing.^11, 17^ Our previous study showed that HOTAIR could also function as a competing endogenous RNA by sponging miR-331-3p in GC.^18^

Recently, numerous lncRNAs have been identified to have a direct role in recruiting PRC2. PRC2, a methyltransferase that is composed of enhancer of zeste homolog 2 (EZH2), suppressor of zeste 12 (SUZ12) and embryonic ectoderm development, can catalyze the di- and trimethylation of lysine residue 27 of histone 3 (H3K27me3), thus modulating gene expression. These lncRNAs epigenetically regulate gene expression through binding to PRC2 in various biological processes, especially in cancer, such us HOTAIR and ANRIL.^13, 16^

Khalil *et al.*^19^, by way of genome-wide RNA immunoprecipitation (RIP) analysis, identified that approximately 20% of the lncRNAs expressed in various cell types are bound to PRC2, including taurine upregulated gene 1 (TUG1). TUG1 was initially detected in a genomic screen for genes upregulated in response to taurine treatment in developing mouse retinal cells. The depletion of TUG1 in the developing mouse eye was found to block retinal development.^20^ In addition, dysregulation of TUG1 could participate in the progression of a variety of tumors.^21, 22, 23^ Our previous study also found that TUG1 could affect cell proliferation through epigenetically regulating HOXB7 by binding to PRC2 in human non-small cell lung cancer.^24^ However, the biological functions of TUG1 in the control of GC tumorigenesis have not been well characterized, which prompted us to explore the role of TUG1 in human GC.

In this study, we found that lncRNA TUG1 was significantly upregulated in GC tissues compared with the corresponding non-tumor lung tissues and may serve as an independent predictor for overall survival in GC. In addition, TUG1 knockdown repressed GC proliferation both *in vitro* and *in vivo*. Further experiments demonstrated that TUG1 was associated with PRC2 and that this association was required for the epigenetic repression of cyclin-dependent protein kinase inhibitors (CKIs), including p15, p16, p21, p27 and p57, thus contributing to the regulation of both the GC cell cycle and proliferation, which may partly account for TUG1-mediated proliferation regulation, thus affecting the proliferation of GC.

## Results

### TUG1 is upregulated in human GC tissues and is positively correlated with deeper tumor invasion depth and advanced TNM stage

**Figure 1:**
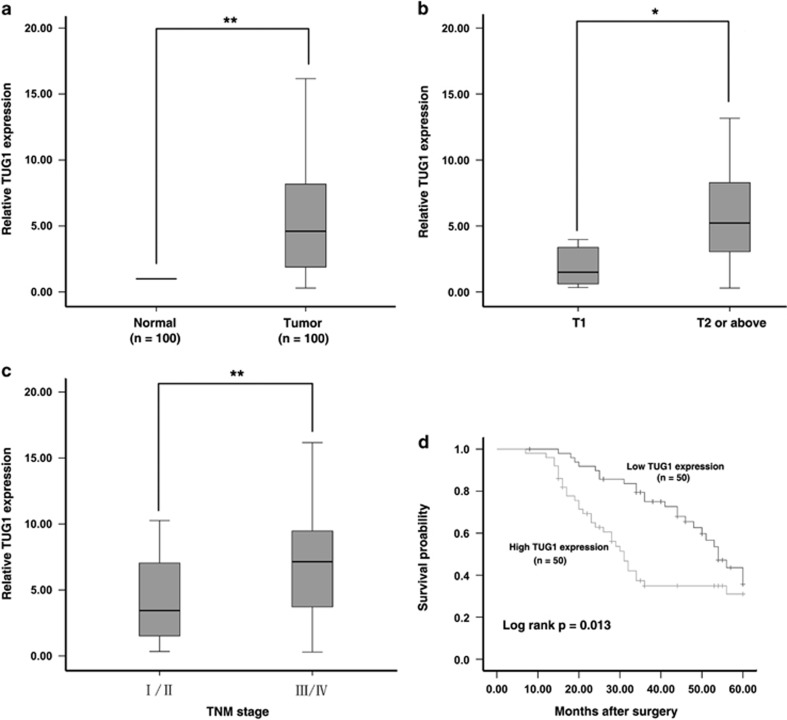
Expression of TUG1 in GC tissues and its clinical parameters. (**a**) Relative expression of TUG1 in GC tissues (*N*=100) compared with the corresponding non-tumor tissues (*N*=100). TUG1 expression was examined using quantitative real-time PCR (qRT-PCR) and normalized to *β*-actin expression. The results are presented as the fold-change in tumor tissues relative to normal tissues. (**b** and **c**) Higher TUG1 was positively correlated with advanced invasion depth and TNM stage. (**d**) Patients with high levels of TUG1 expression showed reduced survival times compared with patients with low levels of TUG1 expression. *P<0.05, **P<0.01

The level of TUG1 was detected in 100 paired GC tissues and adjacent normal tissues using qRT-PCR with normalization to *β*-actin. As shown in Figure 1a, TUG1 expression was significantly upregulated in 85% (85 of 100) of cancerous tissues compared with normal controls (6.0488±6.14159, *P*<0.01). Next, we used a *t*-test to examine the correlation of TUG1 expression level with the clinicopathological features in patients with GC. There was an obvious positive correlation between increased TUG1 levels and deeper tumor invasion depth (6.6585±6.36480 *versus* 2.5940±2.93578, *P*=0.017) and advanced TNM stage (8.3053±7.91956 *versus* 4.3465±3.57530, *P*=0.001) (Figures 1b and c). Furthermore, we divided the samples into high (above the median, *n*=50) and low (below the median, *n*=50) TUG1 expression groups according to the median value of TUG1 levels. A chi-square test was then performed to evaluate the clinicopathological features between the two groups. As shown in Table 1, the TUG1 level was also correlated with tumor invasion depth (*P*=0.002) and TNM stage (*P*=0.009). No relationship between TUG1 expression and other factors, for example, sex (male, female), age (≤60, >60), histological grade (low or undiffer, moderate or high), lymph node metastasis (N0, N1 or above) or distant metastasis (M0, M1), was found in our study.

### Overexpression of TUG1 predicts a poor prognosis and could be regarded as an independent predictor for overall survival of GC

To further evaluate the value of TUG1 in the prognosis of patients with GC, we used a Kaplan–Meier survival analysis and log-rank tests. The median survival time was 54 months in the low TUG1 group, whereas it was 31 months in the high TUG1 group. Overexpression of TUG1 predicted a poor prognosis in patients with GC (*P*=0.013). Univariate analysis identified four prognostic factors: lymph node metastasis (N0, N1 or above), TNM stage (I/II, III/IV), distant metastasis (M0, M1) and TUG1 expression. Multivariate analysis further revealed that TUG1 expression could be regarded as an independent predictor for overall survival in patients with GC (*P*=0.003), as well as TNM stage (*P*=0.019) and lymph node metastasis (*P*=0.001; Table 2).

### TUG1 regulates GC cell proliferation by affecting the cell cycle

**Figure 2:**
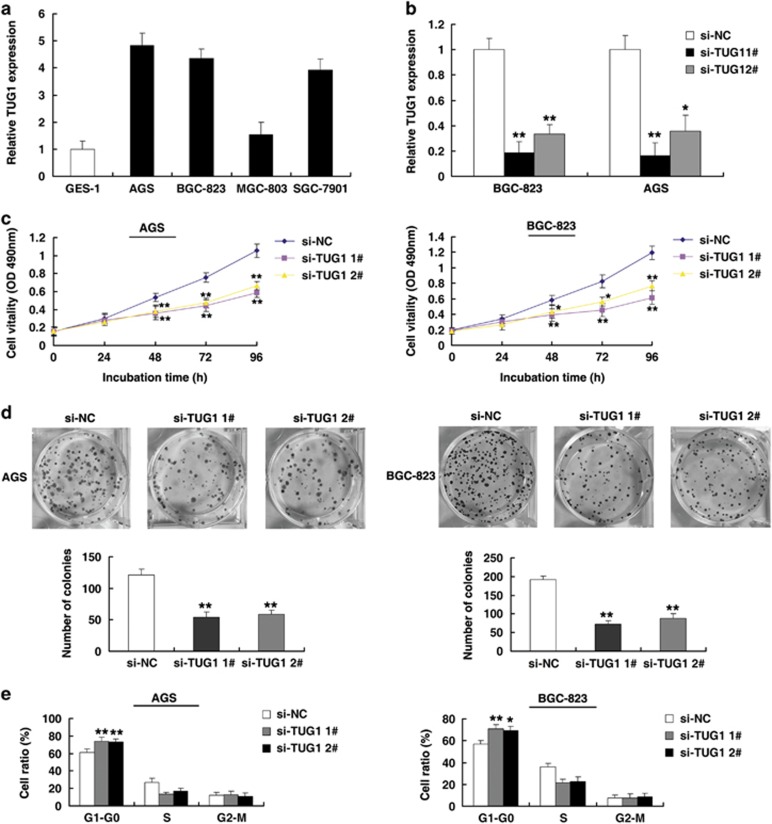
TUG1 regulates GC cell proliferation *in vitro*. (**a**) Analysis of TUG1 expression levels in GC cell lines (AGS, BGC-823, MGC-803 and SGC-7901) compared with a normal gastric epithelium cell line (GES-1) by qRT-PCR. (**b**) The relative expression level of TUG1 in GC cells, transfected with si-NC or si-TUG1 (si-TUG1#1 and #2), was tested using qPCR. (**c**) MTT assays were performed to determine cell proliferation of AGS and BGC-823 cells after transfection of siRNA against TUG1. (**d**) The representative results of colony formation of AGS and BGC-823 cells transfected with siRNA against TUG1. (**e**) At 48 h after transfection, the cell cycle was analyzed by flow cytometry. The bar chart represents the percentage of cells in G1–G0, S, or G2–M phase, as indicated. *P<0.05, **P<0.01

To explore the role of high expression of TUG1 in GC, as shown in Figure 2a, we utilized four GC cell lines expressing higher levels of TUG1 than the normal gastric epithelial cell line (GES-1). Then, TUG1 siRNA was transfected into AGS and BGC-823 cell lines. To avoid off-target effects, we used two effective interference target sequences of TUG1, as previously described.^24^ Q-PCR assays revealed that TUG1 expression was significantly reduced (Figure 2b). The MTT assays showed that knockdown of TUG1 expression significantly inhibited cell proliferation compared with the control cells (Figure 2c). Similarly, the result of colony formation assays revealed that clonogenic survival was significantly decreased following inhibition of TUG1 both in AGS and BGC-823 cell lines (Figure 2d). Next, flow cytometric analysis was performed to further examine the effect of TUG1 on the proliferation of GC cells by altering cell cycle progression. The results revealed that the cell cycle progression of si-TUG1 cells was significantly stalled at the G1–G0 phase compared with cells transfected with si-NC, both in AGS and BGC-823 cell lines (Figure 2e).

### The impact of TUG1 on tumorigenesis *in vivo*

**Figure 3:**
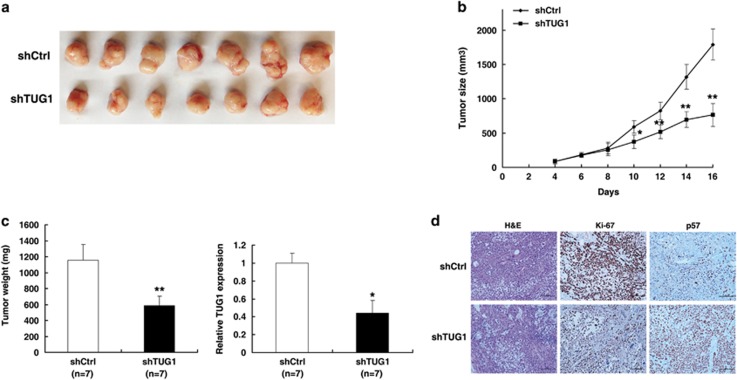
The impact of TUG1 on tumorigenesis *in vivo.* (**a** and **b**) Scramble or shTUG1 was transfected into AGS cells, which were injected into nude mice (*n*=7). The tumor volumes were calculated every 2 days after injection. The bars indicate S.D. (**c**) The tumor weights are shown as means of tumor weights±S.D. qRT-PCR was performed to detect the average expression of TUG1. (**d**) Histopathology of xenograft tumors. The tumor sections underwent H&E staining and IHC staining using antibodies against Ki-67 and p57. Bar, 100*μ*m. Error bars indicate means±S.E.M. *P<0.05, **P<0.01

To further determine whether TUG1 affects tumorigenesis *in vivo*, shCtrl/shTUG1-transfected AGS cells were inoculated into nude mice. Consistent with the *in vitro* results, tumor growth in the shTUG1 group was obviously slower than that in the Scramble group (Figures 3a and b). Up to 16 days after injection, the average tumor weight in the shTUG1 group was significantly lower than that in the control group (Figure 3c). qRT-PCR analysis was performed to detect the average expression of TUG1 in tumor tissues. The results showed that the average level of TUG1 in the shTUG1 group was lower than that in the control group (Figure 3c). Moreover, we also found that the tumors developed from control cells showed stronger Ki-67 expression than tumors formed from shTUG1 and that tumors that developed from shTUG1 cells showed a stronger p57 expression than tumors formed in the control, as detected by IHC analysis (Figure 3d). These data further supported the role of TUG1 in GC cell proliferation.

### TUG1 was required for the epigenetic repression of CKIs by binding to PRC2, thus contributing to the regulation of the GC cell cycle and cell proliferation

**Figure 4:**
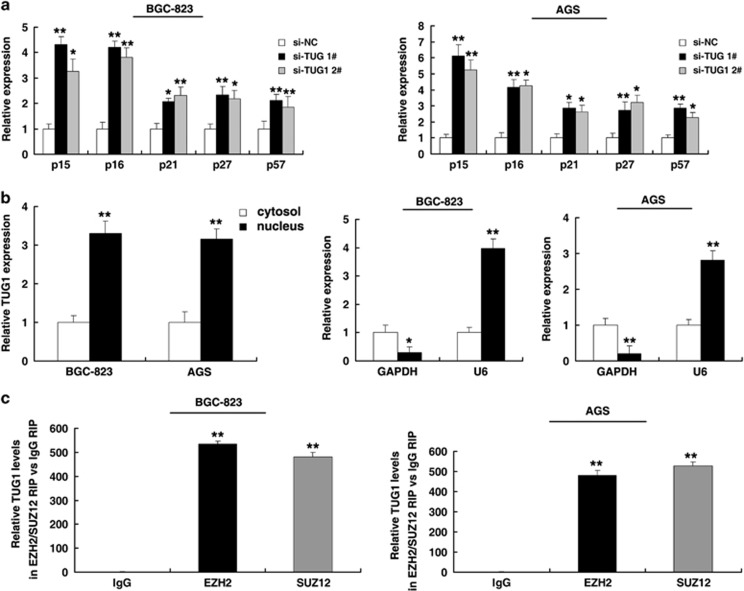
TUG1 is associated with PRC2 in GC. (**a**) The expression of p15, p16, p21, p27 and p57 was determined after knockdown of TUG1 using qRT-PCR. (**b**) TUG1 nuclear localization, as identified using qRT-PCR in fractionated BGC-823 and AGS cells. After nuclear and cytosolic separation, RNA expression levels were measured by qRT-PCR. GAPDH was used as a cytosolic marker, and U6 was used as a nuclear marker. (**c**) RIP experiments were performed, and the coprecipitated RNA was subjected to qRT-PCR for TUG1. The fold enrichment of TUG1 in RIPs is relative to its matching IgG control RIP. *P<0.05, **P<0.01

To explore the fact that TUG1 has a role in G0/G1 arrest, we investigated the expression of CKIs, and the results showed that p15, p16, p21, p27 and p57 were all obviously increased with knockdown of TUG1 (Figure 4a). To further study the mechanism of TUG1 in the regulation of the GC cell cycle, we measured TUG1 expression in nuclear and cytosolic fractions by qRT-PCR. We found a considerable increase in TUG1 expression in the nucleus *versus* the cytosol (Figure 4b), suggesting that TUG1 may have a major regulatory function at the transcriptional level.

To further study the TUG1-associated regulation of GC cell proliferation, we tested whether TUG1 can bind PRC2 in GC cells. As shown in Figure 4c, the endogenous TUG1 was enriched in the anti-EZH2 RIP fraction relative to the input compared with the IgG fraction both in AGS and BGC-823 cell lines. Moreover, using an antibody specific to SUZ12, another member of the PRC2 complex, we also observed that endogenous TUG1 was obviously enriched in the anti-SUZ12 RNA-IP fraction (Figure 4c).

**Figure 5:**
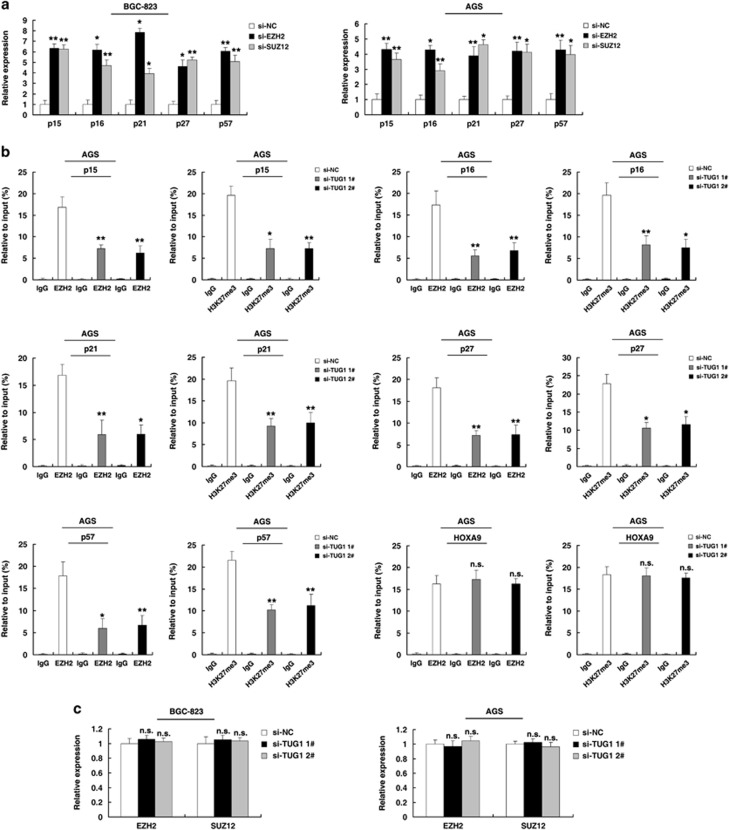
TUG1 is required to target PRC2 occupancy and activity to epigenetically regulate the expression of CKIs, thus regulating GC cell cycle and proliferation. (**a**) The expression of p15, p16, p21, p27 and p57 in BGC-823 and AGS cells, after knockdown of EZH2 and SUZ12. (**b**) ChIP-qPCR of H3K27me3 and EZH2 of the promoter region of the p15, p16, p21, p27 and p57 locus after siRNA treatment targeting si-NC or si-TUG1 in AGS cells. Antibody enrichment was quantified relative to the amount of input DNA. Antibody directed against IgG was used as a negative control. (**c**) The expression of EZH2 and SUZ12 in BGC-823 and AGS cells, after knockdown of TUG1. *P<0.05, **P<0.01

Next, the role of PRC2 in coregulating the suppression of TUG1-suppressed CKIs was investigated by EZH2 knockdown, and both were induced in cells transfected with si-EZH2 (Figure 5a). Similar results were observed for the knockdown of SUZ12 (Figure 5a). To avoid off-target effects, we used an interference target sequence against EZH2 and SUZ12, as studied in a previous article (Supplementary Figure S1A).^25, 26^

To address whether TUG1 is involved in transcriptional repression through the enrichment of EZH2 to target gene promoters, we conducted chromatin immunoprecipitation (ChIP) analysis by TUG1 knockdown. The ChIP assays demonstrated that knockdown of TUG1 decreased the binding of EZH2 and H3K27me3 levels across the p15, p16, p21, p27 and p57 promoters (Figure 5b). As positive controls, no significant change was detected at the promoter of HOXA9, a gene regulated through EZH2.^27^ The levels of EZH2 and SUZ12 were not affected by TUG1-knockdown cells. These results indicated that the decreases in PRC2 chromatin binding and H3K27me3 are mediated by TUG1-knockdown. These results suggest that TUG1 is required to target EZH2 occupancy and works to epigenetically modulate the expression of p15, p16, p21, p27 and p57.

### The roles of EZH2 and p57 in GC

**Figure 6:**
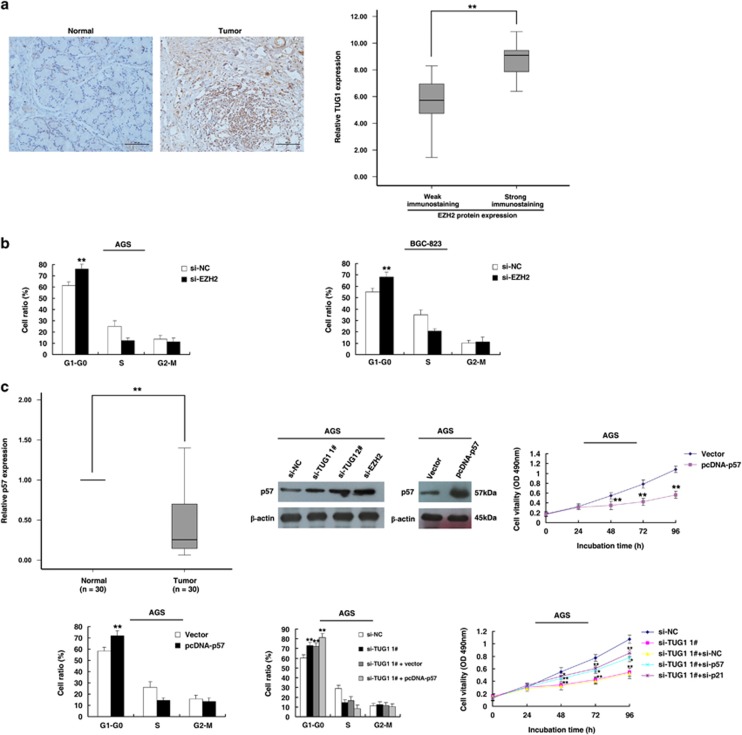
The role of p57 in GC. (**a**) Immunohistochemistry was used to detect the expression of EZH2 protein in 30 pairs of GC with corresponding non-tumor tissues. Bar, 100*μ*m. The immunoreactivity of EZH2 protein in GC tissues showed a statistically significant positive correlation with the relative level of TUG1 expression. (**b**) AGS and BGC-823 cells transfected with si-NC/si-EZH2. Forty-eight hours after transfection, the cells were analyzed using flow cytometry. (**c**) As determined by qRT-PCR assays, the level of p57 was downregulated in 30 pairs of GC tissues. Western blot assays detected the expression of p57 after transfection. AGS cells were transfected with Vector/p57. Forty-eight hours after transfection, the cells were analyzed using MTT assays and flow cytometry. AGS cells were transfected with si-NC/si-TUG1/si-TUG1+vector and transfected with si-TUG1 followed by transfection with pcDNA-p57. After transfection, the cells were stained and analyzed using flow cytometry. MTT analysis of cell proliferation by co-transfection (si-NC, si-TUG1 1#, si-TUG1 1#+si-NC, si-TUG1 1#+si-p57, si-TUG1 1#+si-p21). *P<0.05, **P<0.01

To verify the function of EZH2 in GC, immunohistochemistry was used to detect the expression of the EZH2 protein in 30 pairs of GC with the corresponding non-tumor tissues. All of the tumors showed positive immunostaining for EZH2 protein: 6 of the 30 GC cases (20%) showed weakly positive staining, and 24 GC cases (80%) showed strongly positive staining. In contrast, all of the corresponding non-tumor tissues showed weakly positive immunostaining of EZH2 protein. The representative results are shown in Figure 6a. EZH2 was obviously upregulated in GC tissues. Further analysis showed that the expression of TUG1 was positively correlated with EZH2 protein levels in GC tissues (Figure 6a). Moreover, flow cytometric analysis demonstrated that the cell cycle progression of si-EZH2 cells was stalled at the G1 phase compared with cells transfected with si-NC (Figure 6b).

The functional roles of p15, p16 and p21 have been previously illustrated in GC.^28, 29^ Our previous research suggested that p27 serves as a tumor suppressor in GC.^30^ However, the functional role of p57 in GC remains unclear. First, qRT-PCR was used to detect the expression of p57 in 30 pairs of GC and corresponding non-tumor tissues. As shown in Figures 6c, p57 was obviously downregulated in GC tissues. In addition, the results of western blot assays showed that p57 was obviously increased with the knockdown of TUG1 and EZH2 (Figure 6c). Overexpression of p57 could induce growth inhibition and G1–G0 phase arrest (Figure 6c). Moreover, to further prove the relationships between TUG1 and p57, as shown in Figure 6c, co-transfection of p57 and si-TUG1 promoted TUG1-knockdown-mediated G1–G0 phase arrest. In addition, knockdown of p57 could partly reverse TUG1-knockdown-mediated growth inhibition. TUG1/p21 double knockdown could exert the same effect. The siRNAs could effectively downregulate the expression of p57 and p21 (Supplementary Figure S1B).

## Discussion

To date, the newly discovered lncRNAs have emerged as important factors in cellular development and human diseases. In this study, we found that the average level of TUG1 in GC tissues was significantly higher than in corresponding non-tumor tissues. The high expression level of TUG1 in GC patients was positively correlated with invasion depth and TNM stage. Moreover, high TUG1 expression in GC tissues was associated with a poor prognosis and could be an independent prognostic indicator. These results suggested that TUG1 may have an important role in GC progression. Previous studies found that TUG1 was upregulated in urothelial carcinoma of the bladder, osteosarcoma and esophageal squamous cell carcinoma.^21, 22, 23^ However, our previous study found that TUG1 is downregulated in NSCLC.^24^ This finding is probably because lncRNAs exhibit remarkably tissue-specific expression patterns compared with protein-coding genes and indicates that TUG1 may have a tissue-specific expression pattern.^31, 32^ Moreover, Cao *et al.*^33^ found that TUG1 was upregulated in GC by analysis of lncRNA expression profiles from Gene Expression Omnibus (GEO) in GC. Our results validated the expression results of TUG1 using microarrays with GC tissues and suggested an important role of TUG1 in GC development and progression. In addition, aberrant expression of lncRNAs may be involved in the progression of multiple tumors and can be used as a prognostic indicator.^13, 34, 35^ Our previous studies also showed that the lncRNAs ANRIL, HOTAIR and TINCR could serve as prognostic factors in GC.^16, 18, 36^

Although TUG1 has been studied in a variety of physiological and pathological processes, the possible role of TUG1 in GC remains to be clarified. In our study, the function of TUG1 was investigated by RNA interference (RNAi)-mediated knockdown. As a result, inhibition of TUG1 could promote NSCLC cell proliferation both *in vitro* and *in vivo.* Moreover, the knockdown of TUG1 could induce obvious G0/G1 arrest.

Khalil *et al.*^19^ found that TUG1 could have an important role in the cell cycle of normal cells by binding to PRC2. Our prior study showed that TUG1 also regulated the cell cycle during lung cancer.^24^ In human cancers, overactivation of cyclinD-CDK4/6 kinases or inactivation of the CKIs can result in cell cycle disorders and boost cell proliferation.^37^ The kinase activity of Cdk/cyclin complexes is tightly modulated by CKIs, which serve as brakes to halt cell cycle progression.^38^ In addition, CKIs act as tumor suppressors in various cancers, and aberrant methylation in the CKI gene promoter region has been linked to downregulation of gene expression,^39^ whereas PRC2-mediated histone methylation contributes to the repression of CKIs.^40, 41, 42, 43, 44^ Our results showed that the knockdown of TUG1 could obviously induce the expression of CKIs in an EZH2-dependent manner. Our results explained how CKIs are specifically regulated by PRC2, due in part to TUG1. Many lncRNAs modulate specific genetic loci through recruiting and binding to PRC2 protein complexes, and PRC2-mediated epigenetic regulation has a crucial role in the process of tumor development.^13^

The functional roles of p15, p16 and p21 have been illustrated in GC,^28, 29^ and our previous research showed that p27 serves as a tumor suppressor in GC.^30^ However, the functional role of p57 in GC remains unclear. Our results determined that p57 can serve as a tumor suppressor in GC. Many results have demonstrated that the cell cycle can be regulated by lncRNAs.^45^ These results showed that TUG1 could have a key role in the cell cycle of GC.

In summary, our study identified a TUG1-mediated regulator of the GC cell cycle and cell proliferation. TUG1 may enrich a mechanistic link between lncRNAs and the cell cycle regulation pathway, and TUG1, as a member of PRC2-mediated epigenetic regulation, participates in the occurrence and development of GC. This lncRNA may serve as a target for new therapies in GC.

## Materials and Methods

### Tissue collection and ethics statement

A total of 100 patients analyzed in this study underwent resection of the primary GC at the First Affiliated Hospital of Nanjing Medical University. The study was approved by the Research Ethics Committee of Nanjing Medical University (Nanjing, Jiangsu, China), and written informed consent was obtained from all patients. The clinicopathological characteristics of the GC patients are summarized in Table 1.

### RNA extraction and qRT-PCR analyses

Total RNA was extracted from tissues or cultured cells using TRIzol reagent (Invitrogen, Carlsbad, CA, USA). For qRT-PCR, RNA was reverse transcribed to cDNA by using a Reverse Transcription Kit (Takara, Dalian, China). Real-time PCR analyses were performed with SYBR Green (Takara). The results were normalized to the expression of *β*-actin. The rest of the primers are listed in Supplementary Table 1.

### Cell culture

Four GC cell lines (AGS, SGC-7901, BGC-823 and MGC-803) and a normal gastric epithelial cell line (GES-1) were purchased from the Institute of Biochemistry and Cell Biology of the Chinese Academy of Sciences (Shanghai, China). The cells were cultured in RPMI 1640 or DMEM (GIBCO-BRL) medium supplemented with 10% fetal bovine serum (10% FBS), 100 U/ml penicillin and 100 mg/ml streptomycin in humidified air at 37 °C with 5% CO_2_.

### Cell proliferation assays

Cell proliferation was monitored using the Cell Proliferation Reagent Kit I (MTT) (Roche, Basel, Switzerland). The transfected cells were plated in 96-well plates (3000 cells per well). Cell proliferation was determined every 24 h following the manufacturer's protocol. For the colony formation assay, a certain number of transfected cells were placed into each well of a six-well plate and maintained in media containing 10% FBS for 2 weeks, replacing the medium every 4 days. Colonies were fixed with methanol and stained with 0.1% crystal violet (Sigma-Aldrich, St. Louis, MO, USA) in PBS for 15 min. The colony formation was determined by counting the number of stained colonies. Triplicate wells were measured in each treatment group.

### Western blot assay and antibodies

Cell protein lysates were separated by 10% SDS-polyacrylamide gel electrophoresis, transferred to 0.22-*μ*m NC membranes (Sigma-Aldrich) and incubated with specific antibodies. Autoradiograms were quantified by densitometry (Quantity One software; Bio-Rad, Hercules, CA, USA), and *β*-actin antibody was used as the control. Anti-p57 was purchased from Cell Signaling Technology, Inc (Boston, MA, USA). Antibodies against EZH2 and SUZ12 were purchased from Abcam.

### Flow cytometric analysis

Transfected cells were harvested after transfection by trypsinization. Cells for cell cycle analysis were stained with propidium oxide using the CycleTEST PLUS DNA Reagent Kit (BD Biosciences, Franklin Lakes, NJ, USA) according to the protocol and analyzed by FACScan (BD Biosciences). The percentages of cells in G0–G1, S and G2–M phase were counted and compared.

### Immunohistochemistry

Immunohistochemistry was performed as previously described.^46^ The anti-EZH2 antibody was purchased from Abcam.

### Xenograft study

Five-week-old athymic BALB/c mice were maintained under specific pathogen-free conditions and manipulated according to protocols approved by the Shanghai Medical Experimental Animal Care Commission. AGS cells were transfected with Scramble or shTUG1. After 48 h, the cells were collected and injected into either side of the posterior flank of the nude mouse. Tumor volumes were examined every 2 days when the implantations started to grow. Tumor volumes (length × width^2^ × 0.5) and weights were measured every 2 days in mice from the control (seven mice) or shTUG1 (seven mice) groups. Sixteen days after injection, the mice were killed and the tumor weights were measured.

### Transfection of GC cells

GC cells were transfected with siRNA oligonucleotides with plasmids using Lipofectamine 2000 (Invitrogen, USA) according to the manufacturer's protocol. The nucleotide sequences of siRNA for TUG1 were (siRNA 1# (sense 5′-GCUUGGCUUCUAUUCUGAAUCCUUU-3′, antisense 5′-AAAGGAUUCAGAAUAGAAGCCAAGC-3′); siRNA 2# (sense 5′-CAGCUGUUACCAUUCAACUUCUUAA-3′, antisense 5′-UUAAGAAGUUGAAUGGUAACAGCUG-3′); Negative control siRNA (si-NC) was purchased from Invitrogen (Invitrogen, USA). The p57 and p21 siRNAs were purchased from Santa Cruz (Dallas, TX, USA) (cat. no. sc-35751 and sc-29427). The shTUG1 was cloned into the pENTR™/U6 vector, as previously described.^24^ The sequence of p57 was synthesized and subcloned into a pCDNA3.1 vector (Invitrogen, Shanghai, China). After transfection, the cells were harvested for further studies.

### Subcellular fractionation location

The separation of the nuclear and cytosolic fractions was performed using the PARIS Kit (Life Technologies, Carlsbad, CA, USA) according to the manufacturer's instructions.

### ChIP assays

ChIP assays were performed using the EZ-CHIP KIT according to the manufacturer's instructions (Millipore, Billerica, MA, USA). EZH2 and SUZ12 antibodies were obtained from Abcam. H3 trimethyl Lys 27 antibody was purchased from Millipore. The ChIP primer sequences are listed in Supplementary Table 3. Quantification of immunoprecipitated DNA was performed using qPCR with SYBR Green Mix (Takara). The ChIP data were calculated as a percentage relative to the input DNA using the equation 2^[Input Ct- Target Ct]^ × 0.1 × 100.

### RNA immunoprecipitation

RIP experiments were performed using a Magna RIP RNA-Binding Protein Immunoprecipitation Kit (Millipore) according to the manufacturer's instructions. Antibodies for RIP assays against EZH2 and SUZ12 were purchased from Abcam.

### Statistical analysis

All statistical analyses were performed using SPSS 20.0 software (IBM, SPSS, Armonk, NY, USA). The significant differences between groups were estimated by Student's *t*-test or *χ*2 test, as appropriate. The OS rates were calculated using the Kaplan–Meier method, with the log-rank test applied for comparison. The survival data were evaluated using univariate and multivariate Cox proportional hazards models. Variables with a value of *P*<0.05 in the univariate analysis were used in subsequent multivariate analyses on the basis of Cox regression analyses. Two-sided *P*-values were calculated, and a probability level of 0.05 was chosen for statistical significance.

## Figures and Tables

**Table 2:** Univariate and multivariate analyses of the clinicopathological factors for overall survival in 100 patients with GC

**Risk factors**	**Univariate analysis**			**Multivariate analysis**		
	**HR**	***P*****-value**	**95% CI**	**HR**	***P*****-value**	**95% CI**
TUG1 expression	1.091	<0.001**	1.048–1.137	1.066	0.003**	1.023–1.112
Lymph node metastasis (N0, N1 or above)	2.912	<0.001**	1.609–5.270	2.697	0.001**	1.471–4.946
TNM stage (I/II, III/IV)	2.685	<0.001**	1.573–4.583	2.005	0.019*	1.123–3.579
Distant metastasis (M0, M1)	4.167	0.007**	1.469–11.820	2.52	0.092	0.859–7.393
Histological grade (low or undiffer, middle or high)	0.676	0.15	0.397–1.152			
Age (≤60, >60)	1.182	0.533	0.698–2.001			
Tumor invasion depth (T1, T2 or above)	1.863	0.152	0.796–4.360			
Sex (male, female)	1.473	0.166	0.852–2.546			

*P<0.05. **P<0.01

Abbreviation: HR, hazard ratio

**Table 1:** The clinicopathological factors of GC patients

**Characteristics**	**Expression of TUG1**		***P*****-value**^a^
	**Low**	**High**	
*Sex*			0.84
Male	29	28	
Female	21	22	
*Age*			0.841
≤60	25	24	
>60	25	26	
*Histological grade*			0.161
Low or undiffer	23	30	
Middle or high	27	20	
*Tumor invasion depth (T)*			0.002**
T1	13	2	
T2 or above	37	48	
*Lymph node metastasis (N)*			0.545
N0	23	20	
N1 or above	27	30	
*Distant metastasis (M)*			0.646
M0	48	47	
M1	2	3	
*TNM stage*			0.009**
I/II	35	22	
III/IV	15	28	

a Chi-square test. ** *P*<0.01

## Abbreviations

lncRNA: long noncoding RNA
TUG1: taurine upregulated gene 1
PRC2: polycomb repressive complex 2
GC: gastric cancer
EZH2: enhancer of zeste homolog 2
CKIs: cyclin-dependent protein kinase inhibitors
ChIP: chromatin immunoprecipitation
RIP: RNA immunoprecipitation
