# Supplementary figures and images for: Increased expression of long noncoding RNA TUG1 predicts a poor prognosis of gastric cancer and regulates cell proliferation by epigenetically silencing of p57
Source: Cell Death Dis. 2016 Feb 25;7(2):e2109–. doi: 10.1038/cddis.2015.356 (PMC4849144; doi:10.1038/cddis.2015.356)

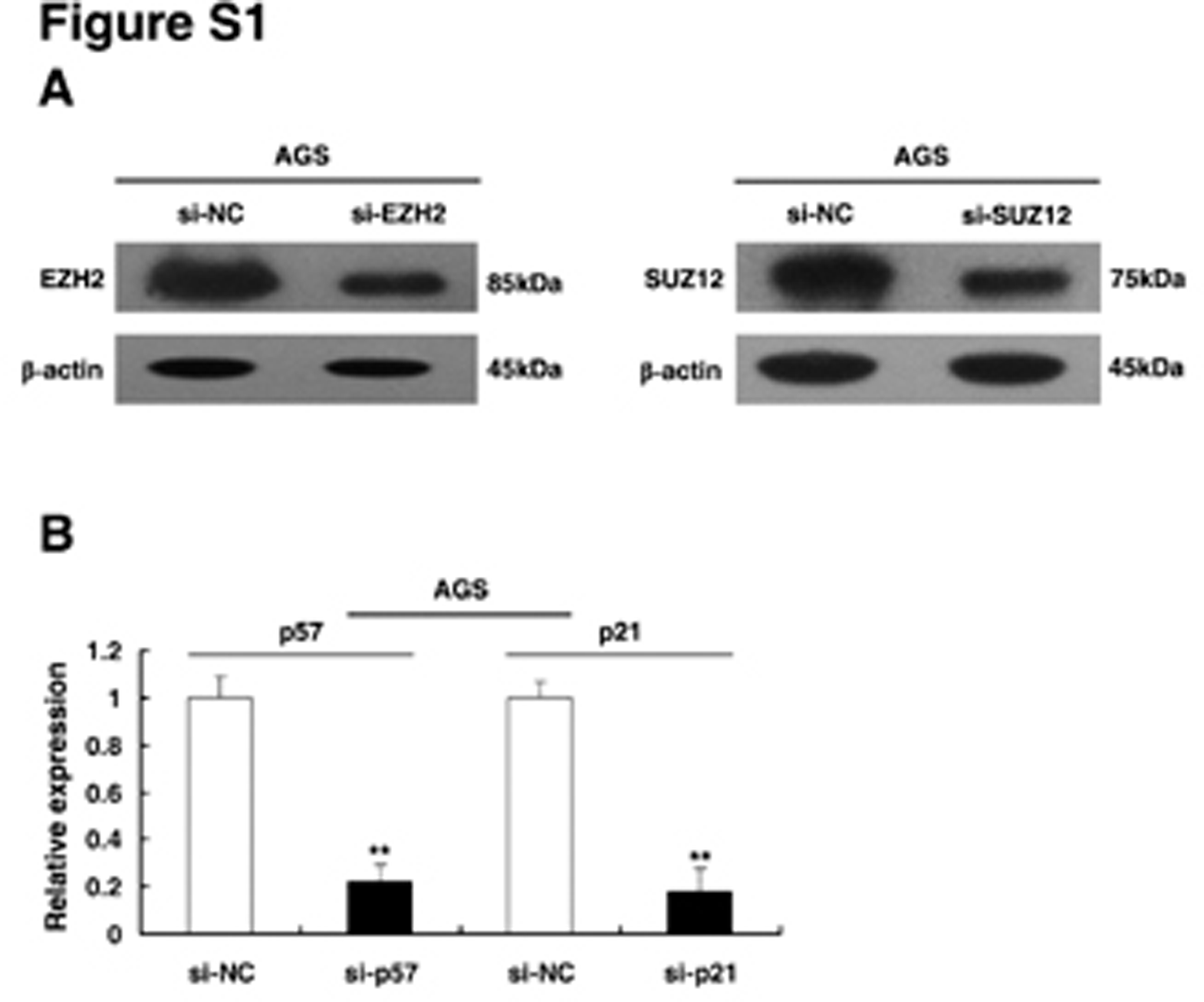

Supplement: Supplementary Figure 1 [file cddis2015356x1.tif]
