# Supplementary material for: Increased expression of long noncoding RNA TUG1 predicts a poor prognosis of gastric cancer and regulates cell proliferation by epigenetically silencing of p57
Source: Cell Death Dis. 2016 Feb 25;7(2):e2109–. doi: 10.1038/cddis.2015.356 (PMC4849144; doi:10.1038/cddis.2015.356)
Supplement: Supplementary Information [file cddis2015356x3.doc]

**Figure S1** (A) Western blot assays detected the expression EZH2 and SUZ12 after si-RNA transfection. (B) The expression of p57 and p21 in AGS cells after knockdown with the respective siRNAs.

**Supplementary Table S1:** The list of primers.
